# Practice changes and infant health risks during the 2022 infant formula shortage: Results of a US healthcare provider survey

**DOI:** 10.1002/ncp.11210

**Published:** 2024-10-02

**Authors:** Marguerite Drowica Sheehan, Diana Orenstein, Leeyu Addisu, Sujata Patil, Devon Kuehn

**Affiliations:** ^1^ ByHeart, Inc New York New York USA; ^2^ Department of Quantitative Sciences Cleveland Clinic Cleveland Ohio USA

**Keywords:** home nutrition support, nutrition, nutrition assessment, nutrition support practice, pediatrics, infant formula, infant formula shortage

## Abstract

**Background:**

In February 2022, an infant formula recall and closing of a major manufacturing center exacerbated a nationwide shortage initiated by COVID‐19–related supply chain disruptions. The effects were far‐reaching, impacting families and healthcare providers across the US.

**Methods:**

A 19‐item web survey was developed to better understand how the infant formula shortage impacted healthcare provider practices, resources needed and those already used, and patient health, including malnutrition. Subjective data on providers' experience were also collected.

**Results:**

Two hundred forty‐one providers responded, primarily registered dietitians (94%) practicing in inpatient/academic hospitals in urban and metropolitan areas. Practice adjustments included increases in patient education (100%), communication with pharmacies/durable medical equipment companies (65%), and visit durations (28%). Feeding adjustments by caregivers included new infant formula (99%), toddler (55%) or homemade (23%) formula, cow's milk (46%) or milk alternatives (32%), formula dilution (41%), and early food introduction (14%). Providers indicated an increase in malnutrition (33%), related diagnoses (including failure to thrive [31%] and deceleration in *z* score [27%]), and associated symptoms. Of the providers who reported malnutrition and related diagnoses, 93% also reported caregiver feeding practices that are generally not recommended.

**Conclusion:**

Providers made practice adjustments to mitigate the consequences associated with formula unavailability and misuse yet saw an increase in malnutrition and related diagnoses or symptoms. Subjectively, providers reported frustration that greater workloads did not result in improved outcomes, contributing to burnout. These data underscore the essentiality of supporting healthcare providers as they guide families in safe infant feeding practices.

**Abbreviations**
AAP: American Academy of Pediatrics
AND: Academy of Nutrition and Dietetics
ASPEN: American Society for Parenteral and Enteral Nutrition
DME: durable medical equipment
FDA: Food and Drug Administration
HCP: healthcare provider
NASPGHAN: North American Society for Pediatric Gastroenterology Hepatology and Nutrition
NEC: necrotizing enterocolitis
PNST: Pediatric Nutrition Screening Tool
SNAP: Supplemental Nutrition Assistance Program
STAMP: Screening Tool for the Assessment of Malnutrition in Pediatrics
WHO: World Health Organization
WIC: Special Supplemental Nutrition Program for Women, Infants, and Children

## INTRODUCTION

On February 17, 2022, the US Food and Drug Administration (FDA) issued a warning to consumers of possible bacterial contamination affecting infant formula produced at the plant of a major infant formula supplier. In response, the manufacturer voluntarily recalled its powdered formula and ceased production at this facility. This event, coupled with COVID‐19–related supply chain issues, led to an unprecedented shortage of infant formula in the US market.^1^ By March 2022, 29% of infant formula was out of stock in all stores. In April 2022, the out‐of‐stock rates reached 40%–50% in 26 states^2^ and >80% in six states.^2^, ^3^ The effects of this shortage were far‐reaching and impacted infants, their families, and healthcare providers (HCPs) across the US. Although there have been publications discussing the impacts of the infant formula shortage on parents, infants, and legislation, to the authors' knowledge, this is the first study investigating actions taken and practice changes by HCPs and how the shortage impacted their practices.

During previous formula and nutrition product shortages, impacts on patient safety and outcomes have been described.^4^ Additionally, HCPs have anecdotally reported impacts on their care of affected infants and increased workloads, leading to feelings of burnout, reduced productivity, and further financial stress on the healthcare sector.^5^, ^6^, ^7^ Burnout has previously been associated with an increased risk of considerable medical errors, reduced productivity, and adverse mental health outcomes.^8^ Therefore, it is pivotal to understand the effects infant formula shortages can have on HCPs.

To gain more insight into HCP experiences during the recent infant formula shortage, an online survey was conducted in collaboration with the American Society for Parenteral and Enteral Nutrition (ASPEN) as part of ASPEN's Malnutrition Awareness Week. The aim of this study was to better understand the changes providers made in their practices, resources used and needed, actions taken by parent(s) or guardian(s), and related impact on patients' health, including malnutrition. Additionally, subjective data were collected to gain a deeper understanding of the challenges faced by providers and their patients' families.

## METHODS

### Survey design

A 19‐item web survey was developed in accordance with the Consensus‐Based Checklist for Reporting of Survey Studies (CROSS) guidelines. In the planning phase, the working group reviewed literature, including surveys of pediatric HCP populations; used existing reporting guidelines; and planned the project timeline. The working group developed a draft of the questionnaire, which was then circulated to a panel of experts chosen for their expertise in pediatric nutrition. The survey was pilot‐tested for clarity, readability, and functionality. After receiving feedback from this expert panel, the working group edited the questionnaire, identifying incomplete items and removing unnecessary items, before circulating it to a second expert panel of pediatric dietitians for final approval. The survey questionnaire can be found in Appendix A. The study was approved by institutional review board before survey distribution.

### Survey content

The multiple‐choice questionnaire was designed to better understand the impact of the infant formula shortage on HCP practice changes, actions taken by parent(s)/guardian(s), and related ramifications on their patients' health, including malnutrition. Additionally, the survey aimed to establish resources HCPs already used before and during the shortage and to identify the resources needed to support HCPs in the future.

### Survey administration and analysis

The survey was created using Typeform. An invitation to participate was distributed by ASPEN to approximately 3000 HCP email subscribers in addition to a private HCP network. The survey was available to respondents during August and September of 2022. Data were analyzed and reported using R‐4.2.1; authors manually contextualized free‐text responses and coded them into categories.

## RESULTS

### Participants' professional degrees, specialties, practice types, and locations

Two hundred forty‐one HCPs across 46 states in the US responded. Ninety‐four percent (94%) were registered dietitians, and the remaining 6% were physicians, nurses, pharmacists, and medical residents. Almost all (99%) worked in urban or metropolitan/metropolitan high‐commuting areas, and 77% were based in inpatient and academic hospitals or clinics. Table 1 provides a detailed summary of the respondent characteristics.

**Table 1:** Characteristics of respondents.

Participant practice information	*n* (%)
Professional degree/specialty	
Dietitian	226 (94.2)
Pharmacist	3 (1.3)
Nurse: hospital based	3 (1.3)
Medical resident	1 (0.4)
Neonatologist	1 (0.4)
Nurse: pediatric office–based nurse practitioner	1 (0.4)
Other physician	1 (0.4)
Pediatric allergist	1 (0.4)
Pediatric gastroenterologist	2 (0.8)
Pediatrician	1 (0.4)
Type of practice^a^	
Hospital inpatient	130 (43.9)
Academic hospital/clinic	96 (32.4)
Durable medical equipment/homecare (other)	29 (10.0)
Community health setting	17 (7.4)
Small outpatient practice	10 (4.5)
Clinic/nonacademic outpatient (other)	9 (3.1)

^a^ Although 241 healthcare providers completed this survey, some practiced in more than one location (ie, working in both outpatient and inpatient settings).

### Formula availability

**Figure 1:**
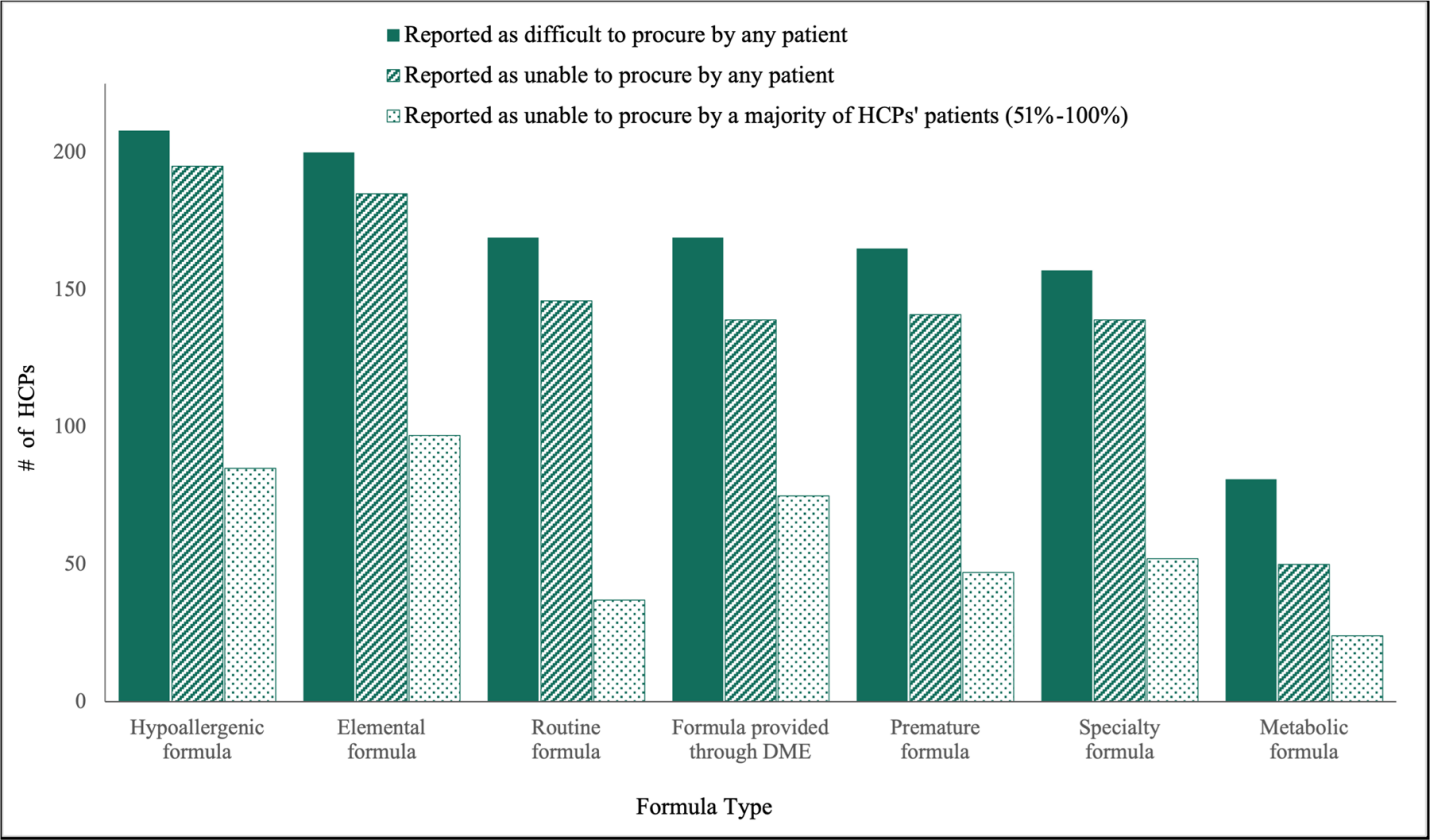
Types of infant formulas that parent(s)/guardian(s) found difficult to procure and the formulas that HCPs' families were unable to procure during the infant formula shortage. Most HCPs reported patients' families had difficulty procuring all formulas (solid bars) and were also unable to procure required formulas (striped bars). Dotted bars represent HCPs who reported most of their patients' families (51%–100%) were unable to procure required formulas. DME, durable medical equipment; HCP, healthcare provider.

Sixty‐seven percent (67%) of providers reported more than half of their patients had difficulty procuring all types of infant formula, with specialty formulas being the most difficult to procure (Figure 1). Of those reporting difficulty procuring infant formula, 93% of HCPs' patients were enrolled in food assistance programs or receiving food assistance, and 71% of families received formula through home healthcare or pharmacies. Of the families receiving formula through home healthcare or pharmacies, 57% required enteral formulas for tube‐fed infants, and 25% required particular specialty formulas, including elemental and hypoallergenic formulas. When asked which populations were impacted the most, 69% of respondents reported that all demographics and socioeconomic status levels within their practicing communities were affected equally.

### Changes to HCP practices

To help parent(s)/guardian(s) navigate the shortage, almost all HCPs (97%) recommended switching to a different formula brand, and 67% recommended checking smaller stores for formula. In addition to encouraging parent(s)/guardian(s) to search for formula on their own, 34% of HCPs also reported an increase in providing formula samples to their patients when able; 25% of HCPs who routinely provided samples before the shortage had to stop because they were unable to access formula samples for their patients. Some HCPs recommended feeding practices that are not considered best practice, such as purchasing formula from abroad (14%), diluting pediatric formula (14%), using whole milk or milk alternatives (9%), or providing a recipe for homemade formula (3%). Table 2 lists all reported recommendations.

**Table 2:** Recommendations HCPs made to parent(s)/guardian(s) during the infant formula shortage.

Recommendations HCPs made to parent(s)/guardian(s) during the infant formula shortage	*n* (%)
Switching to a different brand of formula	233 (96.7)
Checking smaller stores	162 (67.2)
Using local parent groups to identify formula availability	121 (50.2)
Remote parent monitoring of growth between visits	52 (21.6)
Using donor breast milk	44 (18.3)
Purchasing formula from abroad	33 (13.7)
Diluting pediatric formula (for children aged 1–10 years)	33 (13.7)
Vitamin supplementation	33 (13.7)
Using whole milk or alternative milk	21 (8.7)
Providing a recipe for homemade formula	6 (2.5)
Other	21 (8.7)
Provided samples	7 (2.9)
Contacted DMEs directly	7 (2.9)
Other regions in US (shipped)	1 (0.4)
Assistance programs (WIC, SNAP, etc)	2 (0.8)
Mother's breast milk	1 (0.4)
Social media	1 (0.4)
Friends and family	1 (0.4)
Hospital admission as last resort	1 (0.4)

*Note*: *n* = 241 HCPs who answered the question; % refers to percentage of total respondents.

Abbreviations: DME, durable medical equipment; HCP, healthcare provider; SNAP, Supplemental Nutrition Assistance Program; WIC, Special Supplemental Nutrition Program for Women, Infants, and Children.

### Impact on HCP workload, patient education, and resources

HCPs increased the amount of education provided to parent(s)/guardian(s) on the following topics: appropriate infant formula substitutions (89%), formula mixing (77%), and the proper use of infant formula (71%). The most common resource HCPs relied on to make recommendations to parent(s)/guardian(s) was their formal education (73%). In addition to American Academy of Pediatrics (AAP) recommendations (60%), HCPs used resources provided by other medical societies (28%), such as ASPEN (14%) and the North American Society for Pediatric Gastroenterology, Hepatology and Nutrition (NASPGHAN; 5%). Additionally, HCPs looked to published literature (27%) or textbooks (8%), and some used social media for support (12%). When asked which additional resources would be the most beneficial in supporting their knowledge of infant formula and infant malnutrition in the future, HCPs indicated formula comparison charts (72%) would be the most helpful. Following comparison charts, educational and nutrition information on non‐US formulas was the most requested resources (66%). In addition to providing increased patient education, the most reported practice changes were an increase in work and communication with durable medical equipment (DME) companies (65%), coordination of care with community resources (57%), and duration of visits (28%).

Throughout the survey, HCPs were provided the opportunity to comment in free‐text format. Comments were most commonly related to confusion about the shortage, a general lack of governmental and institutional support, increased workload, and eventual feelings of burnout due to the infant formula shortage.

### Actions taken by parent(s)/guardian(s)

When asked about their awareness of feeding practices and changes made by parent(s)/guardian(s) during the shortage, the most reported action taken by parent(s)/guardian(s) was switching to a different formula brand (99%). HCPs also reported the following feeding practices that are generally not recommended: replacing infant formula with toddler formula (55%), cow's milk (46%), or goat milk or other milk alternatives (32%); diluting formula (41%); replacing infant formula with homemade formula (23%); introducing solid foods before 4 months of life (14%); and stopping formula altogether before 12 months of life (50%). All feeding practices are reported in Table 3.

**Table 3:** Feeding practices conducted by parent(s)/guardian(s) as reported by healthcare providers.

Feeding practices conducted by parent(s)/guardian(s)	*n* (%)
Switching to a different brand of formula	237 (99.2)
Replacing infant formula with toddler formula	133 (55.2)
Stopping formula before 12 months of life	121 (50.2)
Replacing infant formula with cow's milk	111 (46.1)
Feeding less formula; diluting formula	98 (40.7)
Replacing infant formula with goat's milk or other alternative milks	77 (32.0)
Replacing infant formula with homemade formula	56 (23.2)
Introducing solid food before 4 months of life	33 (13.7)
Other	9 (3.7)
Juice	3 (1.2)
Continuing to use recalled formula	1 (0.4)
Brought infant to emergency department	1 (0.4)
Increased breastfeeding	2 (0.8)
Parent purchased unpasteurized donor milk off a social media site	1 (0.4)
Brought infant to emergency department	1 (0.4)

*Note*: *n* = 241 HCPs who answered the question; % refers to percentage of total respondents.

### Impact on infant health and malnutrition

The most used nutrition risk screening tools were the Pediatric Nutrition Screening Tool (PNST) (35%), the Screening Tool for the Assessment of Malnutrition in Pediatrics (STAMP) (19%), and those implemented by the HCPs' hospitals or institutions (9%). Although the overall frequency of HCPs using malnutrition screening tools increased, the types of tools used primarily stayed consistent with those used by HCPs before the shortage. Growth charts (94%) and *z* scores (90%) were the most used anthropometric measurements, laboratory studies, or tools used while screening and assessing for malnutrition. Body mass index was also reported as a commonly used malnutrition tool, with 73% of HCPs indicating they routinely used this screening tool before the shortage and continued to do so after February 2022 at the same rate. Additional measurements used were weight (6%), mid‐upper arm circumference (4%), head circumference (3%), triceps skinfolds (2%), and nutrition laboratory studies (8%).

The most used classifications for pediatric malnutrition were provided by medical society consensus statements (51%), World Health Organization (WHO) standards (48%), and Centers for Disease Control and Prevention (CDC) classifications (34%). ASPEN's malnutrition classifications were the next most common (32%), followed by the Academy of Nutrition and Dietetics (AND) malnutrition classifications (24%).

Approximately 52% of HCPs indicated they had diagnosed or treated patients with mild, moderate, or severe malnutrition during the infant formula shortage. Thirty‐three percent reported an increase in malnutrition from typical levels before February 2022 due to insufficient availability or improper use of infant formula (Table S1). In addition to malnutrition, there was an increase in treatment or diagnosis of failure to thrive (32%), deceleration in *z* score (28%), delayed wound healing (3%), or developmental or intellectual delay (4%). Symptoms associated with malnutrition, such as diarrhea (45%) and nausea and vomiting (46%), were reported, along with electrolyte imbalances (30%), the exacerbation of food allergy symptoms (22%), cow's milk protein allergy (20%), and anemia (12%) (Table 4).

**Table 4:** Malnutrition and malnutrition‐related symptoms reported by healthcare providers due to infant formula shortage.

Diagnosis	*n* (%)
Mild malnutrition	118 (49.0)
Moderate malnutrition	89 (36.9)
Severe malnutrition	83 (34.4)
FTT	116 (48.1)
Nausea and vomiting	111 (46.1)
Diarrhea	109 (45.2)
Deceleration in z‐score	108 (44.8)
Electrolyte imbalances; dehydration	73 (30.3)
Exacerbation of food allergy symptoms	52 (21.6)
Cow's milk protein allergies, specifically	48 (19.9)
Anemia	29 (12.0)
Developmental delay	29 (12.0)
Delayed wound healing	18 (7.5)
Seizures	9 (3.7)
Cardiac (heart arrhythmia; hypotension)	4 (1.7)
Other	64 (26.6)
Intolerance	16 (6.6)
Constipation	7 (2.9)
Hyponatremia/hypernatremia	7 (2.9)
Undernutrition; low protein; deficiency	7 (2.9)
Overnutrition; excess nutrients	2 (0.8)
Oral (delay in oral development; dysphagia; aspiration)	4 (1.7)
Gas	2 (0.8)
NEC	2 (0.8)
Reflux	2 (0.8)
Bloody stool	1 (0.4)
Bone health	1 (0.4)
Dumping syndrome	1 (0.4)
Rash	1 (0.4)
Refeeding syndrome	1 (0.4)
Refusal	1 (0.4)
Renal	1 (0.4)
Stress (emotional)	1 (0.4)

*Note*: *n* = 241 HCPs who answered the question; % refers to percentage of total respondents.

Abbreviations: FTT, failure to thrive; NEC, necrotizing enterocolitis.

To evaluate the impact of feeding practices that are not generally recommended, a cross‐tabulation was conducted to examine the relationship between malnutrition treatment and these feeding practices conducted by parent(s)/guardian(s) (Table 5). Of 124 HCPs who reported treating any stage of malnutrition due to the shortage, 95% also reported parent(s)/guardian(s) conducted feeding practices that are generally not recommended (Table 5). Of the 148 HCPs who reported any malnutrition, failure to thrive, deceleration in *z* score, delayed wound healing, or developmental delay, 93% also reported parent(s)/guardian(s) conducted feeding practices that are generally not recommended (Table 5). Lastly, of the 163 HCPs who reported treating any other malnutrition‐related symptoms, 91% also reported parent(s)/guardian(s) conducted feeding practices that are generally not recommended (Table 5).

**Table 5:** Percentage of HCPs who reported malnutrition and related diagnoses who also reported feeding practices that are generally not recommended.

Malnutrition and malnutrition‐related symptoms or illness treated by HCPs	Feeding practices that are generally not recommended		*P* value^a^
	Yes (*n* = 213)	No (*n* = 28)	
Mild, moderate, or severe malnutrition (*n* = 124)			0.001
Yes	118	6	
No	92	20	
Unknown	3	2	
Any malnutrition, failure to thrive, deceleration in *z* score, delayed wound healing, developmental delay (*n* = 148)			0.007
Yes	138	10	
No	73	16	
Unknown	3	2	
Any malnutrition‐related symptom^b^ (*n* = 163)			0.001
Yes	149	7	
No	64	21	
Unknown	0	0	

Abbreviation: HCP, healthcare provider.

^a^ *P* < 0.01; Pearson chi‐squared test; Fisher exact test.

^b^ Excluding mild, moderate, or severe malnutrition; failure to thrive; deceleration in *z* score; delayed wound healing; and developmental delay.

## DISCUSSION

Although there have been publications discussing the impacts of the infant formula shortage on parents, infants, and legislation, this study is the first (to the authors' knowledge) to investigate how the shortage impacted HCPs' practices, in addition to feeding practices conducted by parent(s)/guardian(s) and impacts on infant health outcomes, including malnutrition.^6^, ^9^ Subjective data collection allowed for HCPs' experiences and sentiments to be examined more closely, which led to resource development to help alleviate potential HCP burnout in the case of any future infant nutrition healthcare crises.^10^ The results of this survey indicate the US infant formula shortage resulted in adverse health consequences for infants despite the increased efforts of HCPs to support infants and their parents(s)/guardian(s). HCPs devoted more time to patient visits, patient education on fundamental formula‐related topics, and self‐education yet saw an increase in the diagnosis of malnutrition and malnutrition‐related diagnoses and symptoms.

As expected and seen during other supply chain crises and nutrition shortages, such as the recent COVID‐19 healthcare crisis and shortages related to components of parenteral nutrition therapy over the last decade, an increase in HCP efforts with continued adverse health outcomes for patients contributed to HCP burnout. Specific actions reported by HCPs that contributed to feelings of burnout include lengthening the duration of visits with families, coordinating care with community resources more frequently, increasing communication with DME companies, and dedicating additional hours to planning hospital discharges for any patients with feeding tubes, as they were unable to ensure formula through DMEs would be available once discharged.

HCPs reported that although finding routine formulas was difficult for many patients, hypoallergenic and elemental were the most difficult to procure. HCPs indicated that the shortage particularly impacted patients who relied on DME companies or required specialty formulas. Subsequently, HCPs increased communication with DME companies and coordination of care with community resources to support their patients' parents(s)/guardian(s) during the shortage. These actions align with previously reported data indicating there were minimal alternatives available for infants with severe allergies, intestinal failure, kidney disease, and metabolic disorders.^7^

HCPs also reported that the shortage notably impacted patients who relied on the Special Supplemental Nutrition Program for Women, Infants, and Children (WIC) to procure their formula. This finding is particularly concerning because those purchasing formula using WIC benefits constitute 50% of formula purchasers in the US.^11^ The program requires that WIC state agencies have formula rebate contracts with specific infant formula manufacturers, for which manufacturers must competitively bid. Under this system, only one infant formula brand is available to WIC participants in each state.^12^, ^13^ This system created a particularly fragile link in the nutrition supply chain, making it difficult for families to rely on WIC to find formula.

For parent(s)/guardian(s) who had the option, switching to a different formula was the most reported change to their feeding practices. This action is in line with the most frequently reported recommendation made by HCPs, suggesting that parent(s)/guardian(s) turned to their HCPs as trusted resources during the shortage, which aligns with findings by Cernioglo and Smilowitz.^6^ Although HCPs turned to their own resources, including their formal education and medical societies for guidance, HCPs reported they would have benefited from additional resources such as information on appropriate formula substitutions. This is not surprising given that the most common education topic was formula substitutions, and the most common change to feeding practices was switching to a new formula.

It is important to note that switching formulas was not an option for all families. Although HCPs used trusted sources to guide their recommendations, some providers reported advising parents to dilute pediatric formula, use whole milk or milk alternatives, or make homemade formulas. In extreme cases, HCPs recommended bringing children into the emergency department to access their local hospital's formula supply. Unfortunately, free‐text responses did not provide additional insight or context as to why some HCPs were recommending feeding practices that are not considered best practice. There were likely reasonable explanations for these recommendations; however, they were not captured in this survey. Given the strain on the system and the settings in which most of these HCPs saw patients, HCPs likely weighed the options and provided the best possible recommendations. Further study is warranted to provide resources to support decision‐making in such cases in the future.

Feeding practices that are generally not recommended such as diluting formula, saving leftover formula, and adding cereal to formula during infant formula shortages have previously been reported by Cernioglo and Smilowitz^6^ and Marino et al.^14^ Marino et al. found feeding practices that are generally not recommended were significantly more likely to be reported by parent(s)/guardian(s) with lower incomes compared with those with higher incomes.^14^ In line with those results, almost all survey respondents reported that most of their patients' families were either WIC recipients or enrolled in other food assistance programs. This further underscores the need to provide additional resources to vulnerable communities in the case of future shortages. A key consideration is that respondents of this survey were primarily from urban areas and worked in larger, academic, institutional hospitals; other regions were likely affected, and future studies should explore the impact on other communities.

Most HCPs who reported parent(s)/guardian(s) conducting feeding practices that are generally not recommended also reported treating any stage of malnutrition or malnutrition‐related conditions. The percentage of providers assessing for and treating malnutrition is especially concerning because research indicates that malnutrition, especially in the critical first 1000 days of life, is associated with negative consequences for neurocognitive function, metabolic programming, immune function, and development of the gut microbiome of infants, as well as a predisposition to obesity, diabetes, and cardiovascular disease in adulthood.^15^, ^16^, ^17^ Although not fully understood, these relationships are theorized to result from disruptions in numerous biochemical pathways caused by nutrient deficiencies.^16^ Further studies are needed to elucidate the effect of the shortage on present and future health outcomes.

A considerable limitation of this study is the narrow population of respondents; most were dietitians practicing in urban locations and reported the demographics of their communities were largely homogenous. Additionally, as most respondents were working in a hospital setting, the infants represented may be more at risk for malnutrition and related conditions than what would be the case with a broader group of HCP respondents. Therefore, the results may be skewed toward an urban population and not generalizable to a larger population. Although subjective data indicated many of the respondents were still dealing with the impacts of the shortage at the time of survey distribution, it is important to note this survey was distributed approximately 6 months after the onset of the infant formula shortage. Additionally, empirical data were not collected from affected families or medical records, so these data rely on HCP reports. Developmental delay was included as a malnutrition‐related symptom in the survey because it is considered a potential consequence of pediatric malnutrition. However, given the complexity of developmental delay diagnoses, these reports may not be directly attributable to malnutrition and should be regarded cautiously. Further investigation on the association between infant formula shortages and developmental delays should be conducted in a population of HCPs explicitly trained in diagnosis and treatment. Free‐text responses from HCPs also highlighted the noteworthy impact that the shortage had on infants who require enteral nutrition and other patient populations in need of specialty formulas (toddlers, children, elderly, etc), which this study did not specifically address. When asking about enteral formulas, no further details about the method of use or type of formula were included in the survey. Given the high number of respondents requiring enteral formulas, that information would have provided further insight into this high‐risk population.

A notable strength of this study was the focus on the HCP perspective, particularly the open‐text answers, which allowed for more detail and perspective. Although not explicitly asked in the survey, HCPs provided insight into how their institutions managed shortages and supply chain issues in their free‐text responses. Asking providers about gaps in the available resources allowed for thoughtful resource development to provide tailored, meaningful support for this population. The focus on how the formula shortage led to inappropriate feeding practices and increased malnutrition risk further strengthens the argument for policy change to fortify the infrastructure, as supply chain issues can have long‐term adverse health impacts on our most vulnerable populations.

## CONCLUSION

HCPs made practice adjustments to mitigate malnutrition and illness caused by the misuse of infant formula. Despite providers increasing the amount of education, supported by guidance from medical societies, HCPs reported families conducted feeding practices that posed potential health risks to infants and saw more infants who presented with malnutrition and malnutrition‐related symptoms. Subjectively, providers reported experiencing excess stress and exhaustion due to the lack of governmental and institutional support. Additionally, HCPs reported experiencing burnout, as their increased efforts did not result in improved health outcomes for patients. It is pivotal to continue understanding nutrition shortages' effects on HCPs' practices and experiences, as these effects may impact patient safety and health outcomes. Adding to the established need for policy changes within regulatory and healthcare systems, these data underscore the value of supporting HCPs with thoughtfully designed resources as they guide families in safe infant feeding practices.

## AUTHOR CONTRIBUTIONS

Marguerite Drowica Sheehan, Diana Orenstein, Leeyu Addisu, Sujata Patil, and Devon Kuehn equally contributed to the conception and design of the research. Marguerite Drowica Sheehan, Diana Orenstein, Leeyu Addisu, and Devon Kuehn contributed to the design of the research. Marguerite Drowica Sheehan, Diana Orenstein, Leeyu Addisu, Sujata Patil, and Devon Kuehn contributed to the acquisition, analysis, and interpretation of the data. Marguerite Drowica Sheehan, Diana Orenstein, Leeyu Addisu drafted the manuscript. All authors critically revised the manuscript, gave final approval, and agree to be fully accountable for ensuring the integrity and accuracy of the work.

## CONFLICT OF INTEREST STATEMENT

Marguerite Drowica Sheehan, Diana Orenstein, Leeyu Addisu, and Devon Kuehn are employees of ByHeart. Sujata Patil is a consultant for ByHeart. No funding was received from the American Society of Parenteral and Enteral Nutrition (ASPEN).

## Supporting information

Supporting information.

Supporting information.

## References

[1] USDA continues urgent actions to address infant formula shortage. USDA. Published May 23, 2022. Accessed September 19, 2022. https://www.usda.gov/media/press-releases/2022/05/13/usda-continues-urgent-actions-address-infant-formula-shortage

[2] Datasembly releases latest numbers on baby formula, Datasembly. Datasembly. Published May 10, 2022. Accessed September 19, 2023. https://datasembly.com/news/datasembly-releases-latest-numbers-on-baby-formula/

[3] Paris M . Baby formula shortage worsens to 74% out of stock in US. *Bloomberg*. June 2, 2022. Accessed September 19, 2023. https://www.bloomberg.com/news/articles/2022-06-02/us-baby-formula-shortages-hit-74-despite-biden-action

[4] Holcombe B , Mattox TW , Plogsted S . Drug shortages: effect on parenteral nutrition therapy. Nutr Clin Pract. 2018;33(1):53‐61. 10.1002/ncp.10052 29365360

[5] Mulherin DW , Kumpf V , Shingleton K . Managing nutrition support product shortages: What have we learned? Nutr Clin Pract. 2023;38(1):27‐45. 10.1002/NCP.10927 36309480

[6] Cernioglo K , Smilowitz JT . Infant feeding practices and parental perceptions during the 2022 United States infant formula shortage crisis. BMC Pediatr. 2023;23(1):320. 10.1186/S12887-023-04132-9 37355589 PMC10290398

[7] Abrams SA , Duggan CP . Infant and child formula shortages: now is the time to prevent recurrences. Am J Clin Nutr. 2022;116(2):289‐292. 10.1093/AJCN/NQAC149 35580593 PMC9348970

[8] West CP , Dyrbye LN , Shanafelt TD . Physician burnout: contributors, consequences and solutions. J Intern Med. 2018;283(6):516‐529. 10.1111/JOIM.12752 29505159

[9] Sandoval E , Morris A , Ngo M . A baby formula shortage leaves desperate parents searching for food. *New York Times*. May 10, 2022. Accessed September 19, 2023. https://www.nytimes.com/2022/05/10/us/baby-formula-shortage.html

[10] Infant Formula Shortage Resources. Malnutrition Awareness Week Zoom Room. Impacts of the Infant Formula Shortage: The Health Care Provider's Perspective. October 14, 2022. Accessed June 25, 2024. https://www.nutritioncare.org/uploadedFiles/Documents/Guidelines_and_Clinical_Resources/Infant%20Formula%20Shortage%20Resources.pdf

[11] Doherty T , Coutsoudis A , McCoy D , et al. Is the US infant formula shortage an avoidable crisis? Lancet. 2022;400(10346):83‐84. 10.1016/S0140-6736(22)00984-9 35654081

[12] Dean S . Increasing transparency around WIC infant formula contracts, Food and Nutrition Service. USDA. Published 2022. Updated November 16, 2023. Accessed September 19, 2022. https://www.fns.usda.gov/blog/wic-formula-contracts-transparency

[13] WIC eligibility requirements to bid on state agency infant formula contracts. USDA, Food and Nutrition Service. Published September 8, 2023. Updated August 21, 2024. Accessed September 19, 2023. https://www.fns.usda.gov/wic/requirements-infant-formula-contracts

[14] Marino JA , Meraz K , Dhaliwal M , Payán DD , Wright T , Hahn‐Holbrook J . Impact of the COVID‐19 pandemic on infant feeding practices in the United States: food insecurity, supply shortages and deleterious formula‐feeding practices. Matern Child Nutr. 2023;19(3):e13498. 10.1111/MCN.13498 36949019 PMC10262890

[15] Non AL , Román JC , Gross CL , et al. Early childhood social disadvantage is associated with poor health behaviours in adulthood. Ann Hum Biol. 2016;43(2):144‐153. 10.3109/03014460.2015.1136357 26727037 PMC4977531

[16] Mayneris‐Perxachs J , Swann JR . Metabolic phenotyping of malnutrition during the first 1000 days of life. Eur J Nutr. 2019;58(3):909‐930. 10.1007/S00394-018-1679-0 29644395 PMC6499750

[17] Agosti M , Tandoi F , Morlacchi L , Bossi A . Nutritional and metabolic programming during the first thousand days of life. Pediatr Med Chir. 2017;39(2):157. 10.4081/PMC.2017.157 28673078

