# Supplementary material for: Practice changes and infant health risks during the 2022 infant formula shortage: Results of a US healthcare provider survey
Source: Nutr Clin Pract. 2024 Oct 2;40(2):475–83. doi: 10.1002/ncp.11210 (PMC11879906; doi:10.1002/ncp.11210)
Supplement: Supplementary file 1 — Supporting information. [file NCP-40-475-s002.docx]

**Appendix A**

Supplemental Survey Tool Distributed to United States Health Care Providers:

As a health care provider on the frontline of the national infant formula shortage your input is critical to inform proactive strategy to support infants during times of limited nutrition availability. During this unprecedented time the impact on clinical practice is unknown. We want to hear from you through the completion of a brief survey. The results of this survey will be published and inform the development of resources.

This research is overseen by WCG Institutional Review Board. Your identity will not be collected, and data will be combined for analysis. Your participation in the research is voluntary and there is no compensation for participation. You may choose not to answer any or all questions, and you may stop at any time. The total time to complete the survey should take no more than 20 minutes.

1. **What percentage of your patients/families with infants under the age of 1 year have reported difficulty procuring infant formula since February 2022?**
   - <5%
   - 5-25%
   - 26-50%
   - 51-75%
   - 76-100%

**Of families experiencing difficulty procuring infant formula, approximately what percentage reported being unable to procure:**

- Routine formula: __%
- Hypoallergenic formula: ____ %
- Elemental formula: ____ %
- Metabolic formula: ____ %
- Premature formula: ____ %
- Specialty formula (please specify): ____ %
- Formula provided through Durable Medical Equipment provider: ____ %

**Of families experiencing difficulty procuring infant formula, were any receiving food assistance or enrolled in food assistance programs (i.e., WIC, SNAP, food banks)**

- Yes/No

(If yes, please select all that apply)

- WIC
- SNAP
- FDPIR
- Food banks
- I don’t know
- Other (please specify)
- Were not enrolled in food assistance programs

**Of families experiencing difficulty procuring infant formula, were any receiving formulas through home health care or pharmacies?**

- Yes
  - What was the most frequent reason why these patients needed to receive formula through home health care or pharmacies? (Please specify)
- No

1. **Did you observe that any of the following race/ethnicities were more affected by the formula shortage than others?**

(Please select all that apply)

- White/Caucasian
- Black/African American
- Hispanic/Latino
- Asian
- Multiracial/Multiethnic
- Other (please specify)

**If you chose more than one demographic group above, which was most affected?**

1. **Are you aware of any of the following feeding practices by parents/guardians of your patients during the infant formula shortage?**

(Select all that apply)

- Switching to a different brand of formula
- Formula dilution
- Replacing infant formula with toddler formula
- Replacing infant formula with cow’s milk
- Replacing infant formula with homemade formula (e.g., evaporated milk-based formula)
- Replacing infant formula with goat’s milk or other alternative milks
- Increasing solid food intake
- Stopped formula before 12 months of life
- Introducing solid food prior to 4 months of life
- Other (please enter any other feeding or nutrition behavior changes you became aware of due to limited infant formula availability)

1. **Did you recommend any of the following to families affected by the infant formula shortage?**

(Please select all that apply)

- Diluting pediatric (1-10 year) formula
- Switching to a different brand of formula
- Purchasing formula from abroad
- Checking smaller stores
- Providing a recipe for homemade formula
  - What was the primary nutrient source? (Please specify)
- The use of donor breast milk
- The use of whole milk or alternative milk
  - Please specify type
- Vitamin supplementation
- Remote parent monitoring of growth between routine visits
- Using local parent groups to identify formula availability
- Other (please specify)

1. **Where did you look for guidance to make recommendations?**

(Please select all that apply)

- Medical training (formal education)
- Textbooks
- AAP recommendations
- Research literature/journal articles
- Medical society resources
  - Please specify which medical society
- Social media
- Other (please specify)

1. **Since February 2022, have you increased education of your patient’s families on any of the following?**

(Select all that apply)

- The proper use of infant formula
- Formula mixing
- Appropriate infant formula substitutions
- Nutritional needs of infants
- Signs or symptoms of formula intolerance
- Signs or symptoms of malnutrition
- Other (please specify)

1. **Have you diagnosed or treated patients presenting with any of the following caused by the improper use of infant formula (i.e., dilution, replacement, substitution) since February 2022?**

- Malnutrition (mild), defined as: deceleration across 1 z score line in BMI-for-age OR Weight-for-length
  - Yes – at a rate consistent with rate prior to February 2022 (unchanged)
  - Yes – at a rate increased from before February 2022
  - No
- Malnutrition (moderate), defined as: deceleration across 2 z lines in BMI-for-age OR Weight-for-length
  - Yes – at a rate consistent with rate prior to February 2022 (unchanged)
  - Yes – at a rate increased from before February 2022
  - No
- Malnutrition (severe), defined as: deceleration across 3 z lines in BMI-for-age OR Weight-for-length
  - Yes – at a rate consistent with rate prior to February 2022 (unchanged)
  - Yes – at a rate increased from before February 2022
  - No
- Failure to thrive, defined as: weight consistently below the 3^rd^ to 5^th^ percentile for age and sex, progressive decrease in weight to below the 3^rd^ to 5^th^ percentile, or a decrease in 2 major growth percentiles in a short period
  - Yes – at a rate consistent with rate prior to February 2022 (unchanged)
  - Yes – at a rate increased from before February 2022
  - No
- Deceleration in Z-score (BMI-for-age OR Weight-for-length)
  - Yes – at a rate consistent with rate prior to February 2022 (unchanged)
  - Yes – at a rate increased from before February 2022
  - No
- Delayed wound healing
  - Yes – at a rate consistent with rate prior to February 2022 (unchanged)
  - Yes – at a rate increased from before February 2022
  - No
- Developmental or intellectual delay
  - Yes – at a rate consistent with rate prior to February 2022 (unchanged)
  - Yes – at a rate increased from before February 2022
  - No
- Other (please specify)

1. **In your practice, has there been an increase in diagnosis of any of the following caused by the improper use (i.e., dilution, substitution) or limited supply of infant formula since February 2022?**

(Please select all that apply)

- Exacerbation of food allergy symptoms
- Cow’s milk protein allergies, specifically
- Nausea & Vomiting
- Diarrhea
- Iron deficiency anemia
- Electrolyte imbalances
- Hypotension
- Hypoxemia
- Seizures
- Infections
- Heart arrhythmia
- Other (please specify)

1. **What classifications do you use for pediatric malnutrition locally?**

(Please select all that apply)

- WHO
- CDC
- Society consensus statements
  - Please specify
- Locally defined standards
- Other

1. **Which of the following nutrition risk screening/nutrition awareness tools do you use in your practice?**

(Please select all that apply)

- STAMP (Screening tool for the assessment of Malnutrition in Pediatrics)
- PNST (Pediatric Nutrition Screening Tool)
- StrongKids
- Other (specify)

1. **Which of the following best describes your use of patients’ anthropometric measurements and laboratory studies in nutritional screening/assessment?**

(Please select all that apply)

- Height/length
  - Routinely used before February 2022, continued at the same rate (no change in usage)
  - Routinely used before February 2022, increased use due to shortage (increase in usage)
  - Did not routinely use prior to February 2022, and now routinely using (started due to the shortage)
  - Routinely used prior to February 2022, not using now (stopped due to the shortage)
  - None of the above (please specify)
- Weight
  - Routinely used before February 2022, continued at the same rate (no change in usage)
  - Routinely used before February 2022, increased use due to shortage (increase in usage)
  - Did not routinely use prior to February 2022, and now routinely using (started due to the shortage)
  - Routinely used prior to February 2022, not using now (stopped due to the shortage)
  - None of the above (please specify)
- Mid upper arm circumference (MUAC)
  - Routinely used before February 2022, continued at the same rate (no change in usage)
  - Routinely used before February 2022, increased use due to shortage (increase in usage)
  - Did not routinely use prior to February 2022, and now routinely using (started due to the shortage)
  - Routinely used prior to February 2022, not using now (stopped due to the shortage)
  - None of the above (please specify)
- Head circumference
  - Routinely used before February 2022, continued at the same rate (no change in usage)
  - Routinely used before February 2022, increased use due to shortage (increase in usage)
  - Did not routinely use prior to February 2022, and now routinely using (started due to the shortage)
  - Routinely used prior to February 2022, not using now (stopped due to the shortage)
  - None of the above (please specify)
- Triceps skinfold
  - Routinely used before February 2022, continued at the same rate (no change in usage)
  - Routinely used before February 2022, increased use due to shortage (increase in usage)
  - Did not routinely use prior to February 2022, and now routinely using (started due to the shortage)
  - Routinely used prior to February 2022, not using now (stopped due to the shortage)
  - None of the above (please specify)
- Laboratory studies
  - Routinely used before February 2022, continued at the same rate (no change in usage)
  - Routinely used before February 2022, increased use due to shortage (increase in usage)
  - Did not routinely use prior to February 2022, and now routinely using (started due to the shortage)
  - Routinely used prior to February 2022, not using now (stopped due to the shortage)
  - None of the above (please specify)
- Other (specify)

1. **Which of the following best describes your use of malnutrition screening tools?**

(Please select all that apply)

- BMI
  - Routinely used before February 2022, continued at the same rate (no change in usage)
  - Routinely used before February 2022, increased use due to shortage (increase in usage)
  - Did not routinely use prior to February 2022, and now routinely using (started due to the shortage)
  - Routinely used prior to February 2022, not using now (stopped due to the shortage)
  - None of the above (please specify)
- Percentiles
  - Routinely used before February 2022, continued at the same rate (no change in usage)
  - Routinely used before February 2022, increased use due to shortage (increase in usage)
  - Did not routinely use prior to February 2022, and now routinely using (started due to the shortage)
  - Routinely used prior to February 2022, not using now (stopped due to the shortage)
  - None of the above (please specify)
- Z-scores (weight for age, length for age, HC for age, weight for length)
  - Routinely used before February 2022, continued at the same rate (no change in usage)
  - Routinely used before February 2022, increased use due to shortage (increase in usage)
  - Did not routinely use prior to February 2022, and now routinely using (started due to the shortage)
  - Routinely used prior to February 2022, not using now (stopped due to the shortage)
  - None of the above (please specify)
- Other (specify)

1. **Have you made any of the following changes to your practice since February 2022?**

(Please select all that apply)

- Implemented a new malnutrition screening tool
- Start screening infants for malnutrition routinely
- Lengthened duration of visits to address all parent/patient needs
- Provided psychosocial support to families
- Coordinated care with community resources
- Worked directly with Durable Medical Equipment (DME) providers and pharmacies to identify alternative formulas
- Other (please specify)

1. **Which of the following resources would be of most beneficial for your improving your knowledge of malnutrition in infants?**

(Please select all that apply)

- Short masterclass videos
- Patient handouts
- Case studies
- Patient information packs with video demonstrations
- Videos
- Comparison chart of formulas to easily provide families with alternative options
- Educational and nutritional information on non-US formula
- Other (please specify)

1. **Have you provided samples of formula to families?**

- Routinely provided before February 2022 (unchanged)
- Routinely provided before shortage, and now providing more (increased due to shortage)
- Did not provide prior to shortage, and now routinely providing (started due to the shortage)
- Provided prior to the shortage, not providing now (stopped due to the shortage)
- Did not provide prior to the shortage, and still do not provide (never provided)
- None of the above (please specify)

1. **What is the zip code of your primary practice location?**

(Please enter zip code)

1. **Which of the following is your primary practice location?**

(Please select all that apply)

- Hospital inpatient
- Academic hospital/clinic
- Small outpatient practice
- Community health setting
- Other (please specify)

1. **What best describes your primary practicing professional degree/specialty?**

- Pediatrician
- Neonatologist
- Medical resident
- Pediatric gastroenterologist
- Pediatric allergist
- Other physician (please specify)
- Nurse: Hospital-based
- Nurse: Pediatric office-based nurse practitioner
- Dietitian
- Pharmacist
- Other (please specify)

1. **Is there anything else you would like to share regarding your experience since the national infant formula shortage began?**
