# Supplementary material for: Practice changes and infant health risks during the 2022 infant formula shortage: Results of a US healthcare provider survey
Source: Nutr Clin Pract. 2024 Oct 2;40(2):475–83. doi: 10.1002/ncp.11210 (PMC11879906; doi:10.1002/ncp.11210)
Supplement: Supplementary file 2 — Supporting information. [file NCP-40-475-s001.docx]

**Supplemental Table S1 Revised 08.19.24**

**Table S1.** Effect of Infant Formula Shortage on HCP Reported Frequency of Treatment and Diagnosis of Malnutrition

|  | # of HCPs  n* (%) |
| --- | --- |
| **Mild Malnutrition** | |
| Increased - Treated and diagnosed at a rate increased from before February 2022 | 68 (28.22) |
| Unchanged - Treated and diagnosed at a rate consistent with rate prior to February 2022 | 50 (20.75) |
| Did not treat mild malnutrition before or during the infant formula shortage | 117 (48.55) |
|  | |
| **Moderate Malnutrition** | |
| Increased - Treated and diagnosed at a rate increased from before February 2022 | 43 (17.84) |
| Unchanged - Treated and diagnosed at a rate consistent with rate prior to February 2022 (unchanged) | 46 (19.09) |
| Did not treat moderate malnutrition before or during the infant formula shortage | 142 (58.92) |
|  | |
| **Severe Malnutrition** | |
| Increased - Treated and diagnosed at a rate increased from before February 2022 | 35 (14.52) |
| Unchanged - Treated and diagnosed at a rate consistent with rate prior to February 2022 (unchanged) | 48 (19.92) |
| Did not treat severe malnutrition before or during the infant formula shortage | 149 (61.38) |
|  | |
| **Mild, Moderate or Severe Malnutrition** | |
| Treated and diagnosed at a rate consistent with or increased from before February 2022 | 124 (51.45) |
| Did not treat malnutrition before or during the infant formula shortage | 112 (46.47) |

*Total n-value varies due to non-responses in each section.
